# Genetic variation and expression levels of tight junction genes identifies association between *MAGI3* and inflammatory bowel disease

**DOI:** 10.1186/s12876-017-0620-y

**Published:** 2017-05-25

**Authors:** Elisabeth Norén, Sven Almer, Jan Söderman

**Affiliations:** 1 Department of Medicine, Karolinska Institutet, Solna, Stockholm Sweden; 2 Division of Medical Diagnostics, Region Jönköping County, Jönköping, Sweden; 3 GastroCentrum, Karolinska University Hospital, Solna, Stockholm Sweden; 4 Department of Clinical and Experimental Medicine, Faculty of Health Sciences, Linköping University, Linköping, Sweden

**Keywords:** Inflammatory bowel disease, Tight junctions, Single nucleotide polymorphism, Genetic predisposition, Gene expression

## Abstract

**Background:**

Inflammatory bowel disease (IBD) is associated with increased intestinal permeability, which involves paracellular passage regulated through tight junctions (TJ). The aim of the study was to investigate single nucleotide polymorphisms (SNP) located in genes encoding interacting TJ proteins and corresponding expressions, in relation to IBD.

**Methods:**

Allelic associations between TJ-related genes (*F11R*, *MAGI1*, *MAGI2*, *MAGI3*, *PARD3*, *PTEN*, and *TJP1*) and IBD, Crohn’s disease (CD), or ulcerative colitis (UC) were investigated. *PTPN22* was included since it’s located in the same genetic region as *MAGI3*. Gene expression levels were investigated in relation to genotype, inflammatory status, phenotype, and medical treatment.

**Results:**

The two strongest allelic associations were observed between IBD and SNPs in *MAGI2* (rs6962966) and *MAGI3* (rs1343126). Another *MAGI3* SNP marker (rs6689879) contributed to increased ileal *MAGI3* expression level in non-IBD controls. Furthermore, association between inflammation and decreased expression levels of *MAGI3*, *PTEN*, and *TJP1* in colonic IBD as well as UC mucosa, and between inflammation and increased expression of *PTPN22* in colonic IBD mucosa, was observed.

**Conclusions:**

Our findings lend support to a genetic basis for modulation of intestinal epithelial barrier in IBD, and we have identified *MAGI3* as a new candidate gene for IBD.

**Electronic supplementary material:**

The online version of this article (doi:10.1186/s12876-017-0620-y) contains supplementary material, which is available to authorized users.

## Background

Inflammatory bowel disease (IBD), including Crohn’s disease (CD) and ulcerative colitis (UC), are complex diseases thought to result from loss of homeostasis between the intestinal microbial milieu, the immune system, and a genetic predisposition [1]. Genetic association in IBD has been the focus of much research [2, 3] leading to the recent identification of a large number of susceptibility loci [3–5]. Still, the hitherto identified genetic associations only account for a small fraction of the heritability of CD and UC [3].

IBD has been linked to increased paracellular permeability [6, 7]. The intestinal permeability is further increased in both CD patients and their relatives, indicating underlying hereditary factors [8]. However, it remains controversial whether this affected permeability is primary, caused by genetic factors, and/or secondary to inflammation or environmental factors. The tight junction (TJ) structure is critical for the permeability properties of the intestine [9] and several studies provide support for a genetic basis in increased permeability [10].

Wapenaar et al. [11] have described an association between celiac disease and single nucleotide polymorphism (SNP) markers in *MAGI2* and *PARD3*. This *MAGI2* marker was also associated with UC [11]. Additionally, McGovern et al. [12] identified a link between genetic variation in *MAGI2* and both CD and UC.

**Fig 1:**
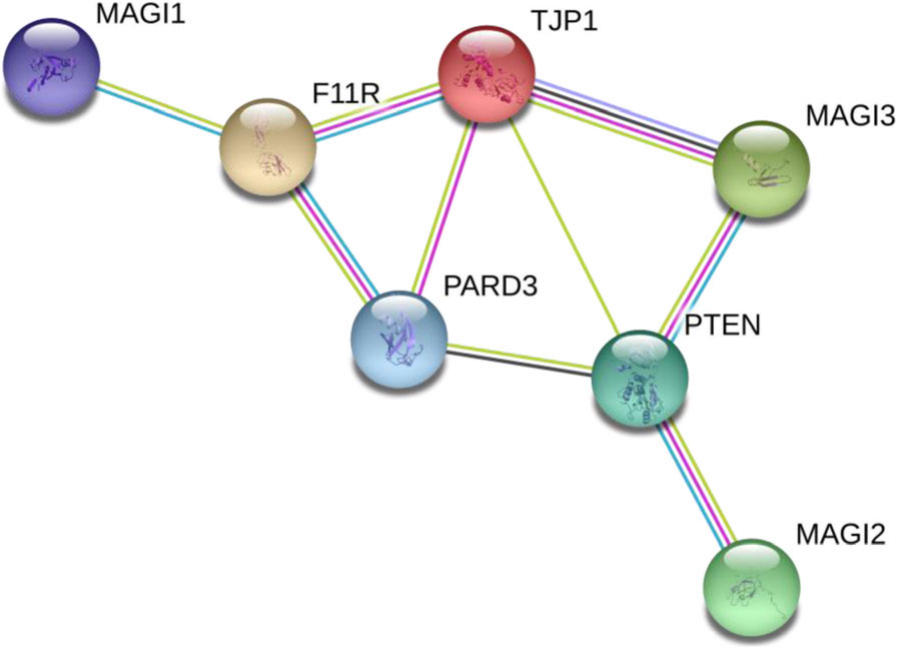
A network consisting of seven TJ genes (*F11R*, *MAGI1*, *MAGI2*, *MAGI3*, *PARD3*, *PTEN*, and *TJP1*) identified using the STRING search tool [14, 15]

**Fig 2:**
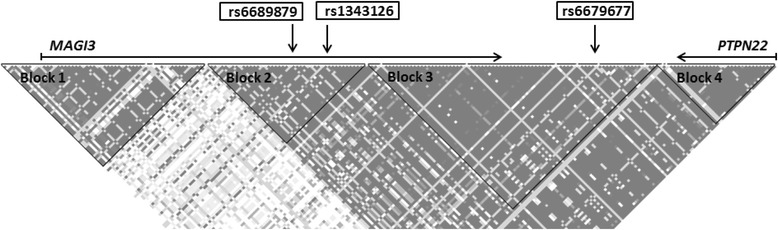
Illustration of the genetic region of *MAGI3* and *PTPN22*. SNP data were obtained from the HapMap database (available at: www.hapmap.org; HapMap Data Rel 28 Phase II + III, August10) and analyzed using Haploview version 4.2 (available at www.broad.mit.edu/haploview) with blocks defined according to Gabriel et al. [17]

The aim of this study was to investigate relations between IBD and *MAGI2* and *PARD3*, as well as other TJ genes (*F11R*, *MAGI1*, *MAGI3*, *PTEN*, and TJP1) encoding products interacting with each other (Fig. 1) [13–15]. Additionally, *PTPN22* was included since it is located in the same genetic region as *MAGI3* (Fig. 2) and has previously been described in relation to IBD [5, 16]. To gain a better understanding of the pathogenic mechanism of IBD we further analyzed ileal and colonic gene expression in relation to genotype, inflammatory status, phenotype, and ongoing medical treatment.

## Methods

### Study subjects

Genetic association was investigated in adult Swedish patients with IBD (138 and 157 patients with CD or UC, respectively) and controls from an anonymized regional DNA bank consisting of randomly selected individuals (*n* = 423) living in the southeastern part of Sweden was used after verbal permission from Prof. Peter Söderkvist (Department of Clinical and Experimental Medicine, Linköping University, Linköping, Sweden) (Table 1, subgroup 1).

**Table 1:** Summary of study participants in subgroup 1

Cohort and disease^a^	Number of individuals	Number of women
Control subjects	423	223 (52.7%)
CD cases	138	72 (52.2%)
UC cases	157	70 (44.6%)

*CD* Crohn’s disease, *UC* ulcerative colitis

^a^Control subjects are from an anonymized regional DNA bank consisting of randomly selected individuals living in the southeastern part of Sweden

A second Swedish cohort (Table 2, subgroup 2), from which both DNA and RNA were available, was recruited to follow up the case–control study of subgroup 1. Blood samples and intestinal biopsy specimens were obtained from adult IBD patients and non-IBD controls. Each intestinal biopsy was categorized as inflamed or non-inflamed based on a compound evaluation of endoscopic findings assessed by one experienced endoscopist (S.A.) and routine histopathologic assessment for inflammation. Only biopsies with concordant results were analyzed further. In total the study included biopsies from 52 Swedish IBD patients (42 inflamed biopsies and 55 non-inflamed biopsies), including 21 CD patients (16 inflamed biopsies and 24 non-inflamed biopsies), 29 UC patients (24 inflamed biopsies and 29 non-inflamed biopsies), 2 IBD-type unclassified (IBDU; 2 inflamed biopsies and 2 non-inflamed biopsies), and 33 non-inflamed non-IBD controls (86 biopsies).

**Table 2:** Summary of study participants in subgroup 2

Disease and subgroups^a^	Number of individuals (women)	Number of non-inflamed biopsies	Number of inflamed biopsies
IBD	52 (29)		
Ileum		24	7
Colon		31	35
CD	21 (13)		
Ileum		11	7
Colon		13	9
UC	29 (15)		
Ileum		12	0
Colon		17	24
Non-IBD	33 (20)		
Ileum		24	0
Colon		62	0

*IBD* inflammatory bowel disease, *CD* Crohn’s disease, *UC* ulcerative colitis, *IBDU* IBD-type unclassified, *non*-*IBD* non-inflamed non-IBD controls

^a^The total IBD group included CD (*n*=21), UC (*n*=29), and IBDU (*n*=2) patients

### SNP selection for genetic association studies

TJ-related genes (*F11R*, *MAGI1*, *MAGI2*, *MAGI3*, *PARD3*, *PTEN*, and *TJP1*) encoding products interacting with each other [13, 14] were investigated (Fig. 1). Additionally, *PTPN22* was included since it is located in the same genetic region as *MAGI3* (Fig. 2) and has previously been described in relation to IBD [5, 16]. All SNP markers are given in Additional file 1: Table S1.

SNP markers (minor allele frequency ≥10%, pair-wise *r* ^2^ ≥ 0.8) of *F11R*, *MAGI1*, *MAGI3*, and *PTEN* were selected using SNPbrowser Software version 4.0 (Applied Biosystems, Foster City, CA). SNP markers for *MAGI1* and *MAGI3* were limited to exons and exon-intron boundaries, due to the large size of these genes. All SNP markers in the genetic region of *MAGI2* [11, 12], *MAGI3*-*PTPN22* [5], *PARD3* [11], *TJP1* [11], and an additional marker for *MAGI1* (rs9880851) [11] were selected from the literature.

### Definition of genetic blocks of *MAGI3* and *PTPN22*

Blocks of genetic linkage disequilibrium in the region of *MAGI3* and *PTPN22* were identified using data from HapMap (available at: www.hapmap.org; HapMap Data Rel. 28 Phase II + III, August10) (Fig. 2). The blocks were visualized using Haploview and the algorithm by Gabriel et al. [17] based on 95% confidence bounds on D’ (Haploview version 4.2, available at: www.broad.mit.edu/haploview).

### Genotyping

DNA was isolated from buffy coat or whole blood (EDTA blood) using the MagNA Pure LC DNA Isolation Kit and MagNA Pure extraction robot (Roche, Basel, Switzerland). In patients where no DNA from blood was available, the genotyping was performed using DNA isolated from intestinal biopsies (isolation previously described [18]). Allelic discrimination was carried out using either the TaqMan OpenArray system or real-time PCR, as well as TaqMan SNP genotyping assays (Applied Biosystems, Foster City, CA) (Additional file 1: Table S1).

Allelic discrimination using TaqMan OpenArray Genotyping Plates, TaqMan OpenArray Genotyping Master Mix, GeneAmp PCR System 9700, and OpenArray NT Imager (Applied Biosystems) was in accordance with the manufacturer’s recommendation. Genotype data were analyzed using OpenArray SNP Genotyping Analysis Software version 1.0.3 and TaqMan Genotyper Software v.1.3 (Applied Biosystems).

The allelic discrimination using real-time PCR was performed in a total reaction volume of 10 μL, consisting of TaqMan SNP genotyping assays and TaqMan Genotyping Master Mix using either the 7500 Fast Real-Time PCR System (Applied Biosystems) or the CFX96 Real-time System, C1000 Touch Thermal Cycler (Bio-Rad Laboratories Inc., Hercules, CA). The genotype data was analyzed using 7500 Software version 2.0.6 (Applied Biosystems) or Bio-Rad CFX Manager 3.1 (Bio-Rad Laboratories Inc.).

### Gene expression analysis

For genes with a significant genetic association to IBD, CD, or UC (in this study), gene expression was analyzed in relation to genotype, inflammatory status, phenotype, and ongoing medical treatment.

RNA purification was performed as described previously [18]. cDNA was synthesized from 2 μg RNA in a total volume of 40 μL using High Capacity cDNA Reverse Transcription kit with RNase Inhibitor according to the manufacturer’s instructions (Invitrogen, Carlsbad, CA). The cDNA was diluted to 25 ng/μL using 0.1 × TE buffer and stored at −80 °C until analysis.

Gene expression of *F11R* (Hs00170991_m1), *MAGI2* (Hs00202321_m1), *MAGI3* (Hs00326365_m1), *PTEN* (Hs02621230_s1), *PTPN22* (Hs01587518_m1), and *TJP1* (Hs01551861_m1) was analyzed using TaqMan Gene Expression Assay (Applied Biosystems), TaqMan Universal Mastermix (Applied Biosystems), and a 7500 Fast Real-Time PCR System (Applied Biosystems). Each individual reaction contained 10 ng cDNA in a total reaction volume of 20 μL.

Threshold cycle (C_T_) values were established (ExpressionSuite Software Version 1.0.3; Applied Biosystems) and normalized to the average of selected reference genes (*CASC3* (Hs00201226_m1), *UBA52* (Hs03004332_g1), and *POP4* (Hs00198357_m1) [18]) generating delta-C_T_ (ΔC_T_) values. Relative quantification (RQ) values were further established, according to Livak et al. [19], by relating the ΔC_T_ value to the sample with the lowest gene expression for each gene (using Microsoft Office Excel; Microsoft Corporation, Redmond, WA). Group differences in gene expression were calculated as a fold change (fc) based on RQ values.

### Statistical analysis

Allelic odds ratios (OR) and *p*-values, based on chi-squared (*χ*2) tests, were calculated using JMP Genomics 6.0 (JMP Genomics 6.0; SAS Institute Inc., Cary, NC). Deviation from Hardy-Weinberg equilibrium was analyzed using the exact test implemented in Haploview version 4.2 (available at: http://www.broad.mit.edu/haploview). For these statistical tests, *p* < 0.05 was considered significant.

Genes with significant association to IBD, CD, or UC were further analyzed. Group differences in gene expression were investigated using Kruskal-Wallis ANOVA or Mann–Whitney *U* test (Statistica 12; StatSoft Inc., Tulsa, OK). Gene expression levels were investigated in relation to genotype using Spearman’s rank correlation test (Statistica version 12.7; StatSoft Inc., Tulsa, OK) and also in relation inflammatory status, phenotype, and ongoing medical treatment using logistic regression (Statistica version 12.7; StatSoft Inc.). The analyses of the group differences of gene expressions as well as the logistic regressions were performed using ΔC_T_ values. Box plots were created using Statistica version 12.7 (StatSoft Inc.). A Bonferroni adjusted *p* < 0.008 (based on the number of analyzed genes; *n* = 6) was considered significant for statistical analysis based on gene expression levels.

### Ethical considerations

This study was approved by the ethics committee of Linköping University (dnr. M35-07, dnr. 2011/201-31). Written and informed consent were obtained from all study participants.

## Results

### Genetic association – case–control approach

Of the 64 SNP markers, twelve were excluded due to failed genotyping or absence of Hardy-Weinberg equilibrium (Additional file 1: Table S1). The two strongest associations were observed between IBD overall and SNP markers in *MAGI2* (rs6962966; susceptibility allele A; *p* = 0.004) and *MAGI3* (rs1343126; susceptibility allele T; *p* = 0.004) (Additional file 2: Table S2). These two markers were also associated with CD (*p* = 0.008 and *p* = 0.051, respectively) and UC (*p* = 0.045 and *p* = 0.011, respectively) individually, even if the association between CD and the *MAGI3* SNP marker was borderline significant. Associations were also observed between IBD overall and *MAGI3* (rs12119076, *p* = 0.022), as well as between UC and *F11R* (rs7546890, *p* = 0.043), *MAGI2* (rs7803276, *p* = 0.031), *MAGI3* (rs6689879, *p* = 0.043), *PTEN* (rs1234226, *p* = 0.046), and *TJP1* (rs260526, *p* = 0.010).

### Gene expression

Biopsies from different colonic segments (cecum, ascending colon, transverse colon, descending colon, sigmoid colon, and rectum) were treated as biological replicates since all genes except *TJP1* were expressed at equal levels at these locations in non-IBD controls (data not shown). *TJP1* showed slightly higher expression in the sigmoid colon compared to ascending colon (*p* = 0.006; fc = 1.16). The biopsy specimens from ileum and colon were analyzed separately.

#### Gene expression versus genotype

Carriage of at least one UC susceptibility allele (C) of *MAGI3* rs6689879 contributed to increased *MAGI3* expression level in ileal non-IBD mucosa (*p* = 0.002; fc = 1.7) (Table 3). No other SNP markers contributed significantly to the gene expression level of corresponding genes.

**Table 3:** For genes with a significant genetic association to IBD, CD, or UC, gene expression (ΔCt values) was analyzed in relation to genotype, in intestinal biopsies from the different subgroups, using Spearman’s rank correlation test

		Non-IBD		Non-inflamed IBD		Inflamed IBD	
Gene	Gene SNP^a^	Ileum	Colon	Ileum	Colon	Ileum	Colon
		*p*-value^b^	*p*-value^b^	*p*-value^b^	*p*-value^b^	*p*-value^b^	*p*-value^b^
*F11R*	rs7546890 (T)	0.144 (5, 14, 5)	0.112 (6, 19, 8)	0.167 (5, 13, 6)	0.541 (8, 15, 6)	0.140 (1, 4, 1)	0.347 (7, 16, 5)
*MAGI2*	rs7803276 (C)	0.009 (10, 8, 6)	0.603 (11, 15, 7)	0.604 (5, 11, 8)	0.029 (8, 14, 7)	0.637 (3, 2, 1)	0.285 (6, 13, 9)
	rs6962966 (A)	0.797 (11, 8, 5)	0.657 (14, 11, 8)	0.266 (5, 11, 8)	0.868 (6, 16, 7)	0.954 (3, 1, 2)	0.817 (6, 13, 9)
*MAGI3*	rs6689879 (C)	0.002 (4, 7, 13)	0.050 (5, 10, 18)	0.497 (1, 8, 15)	0.785 (2, 13, 14)	0.021 (0, 3, 3)	0.355 (1, 13, 14)
	rs1343126 (T)	1.000 (0, 14, 10)	0.916 (0, 16, 17)	0.044 (1, 15, 8)	0.737 (2, 14, 13)	0.594 (1, 3, 2)	0.780 (0, 18, 10)
	rs12119076 (C)	0.978 (0, 13, 11)	0.777 (0, 15, 18)	0.044 (1, 15, 8)	0.737 (2, 14, 13)	0.594 (1, 3, 2)	0.780 (0, 18, 10)
	rs6679677^d^	0.266 (18, 6, 0)	0.018 (25, 8, 0)	0.477 (22, 2, 0)	0.834 (26, 3, 0)	NA^c^ (6, 0, 0)	0.047 (25, 3, 0)
*PTEN*	rs1234224 (G)	0.554 (2, 12, 10)	0.458 (3, 16, 14)	0.851 (1, 9, 14)	0.772 (2, 15, 12)	NA^c^ (0, 0, 6)	0.695 (3, 14, 11)
*PTPN22*	rs6689879 (C)	0.697 (4, 7, 13)	0.509 (5, 10, 18)	0.634 (1, 8, 15)	0.129 (2, 13, 14)	0.854 (0, 3, 3)	0.764 (1, 13, 14)
	rs1343126 (T)	0.227 (0, 14, 10)	0.025 (0, 16, 17)	0.870 (1, 15, 8)	0.822 (2, 14, 13)	0.908 (1, 3, 2)	0.963 (0, 18, 10)
	rs12119076 (C)	0.555 (0, 13, 11)	0.089 (0, 15, 18)	0.870 (1, 15, 8)	0.822 (2, 14, 13)	0.908 (1, 3, 2)	0.963 (0, 18, 10)
	rs6679677^d^	0.128 (18, 6, 0)	0.183 (25, 8, 0)	0.147 (22, 2, 0)	0.291 (26, 3, 0)	NA^c^ (6, 0, 0)	0.745 (25, 3, 0)
*TJP1*	rs260526 (G)	0.285 (21, 3, 0)	0.052 (29, 4, 0)	0.582 (21, 3, 0)	0.396 (24, 5, 0)	NA^c^ (6, 0, 0)	0.517 (22, 5, 1)

*IBD* inflammatory bowel disease, *CD* Crohn’s disease, *UC* ulcerative colitis

^a^The susceptibility allele is shown in parentheses. ^b^Number of individuals in each genotype group is shown in parentheses (individuals homozygous for the susceptibility allele, heterozygous individuals, and individuals homozygous for the non-susceptibility allele). ^c^NA; not applicable. ^d^The susceptibility allele (C) is defined from Jostins *et. al* [5]

#### Gene expression versus inflammation

**Fig 3:**
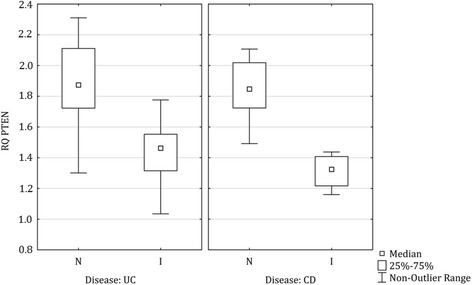
Box Plot illustration of *PTEN* expression level in colonic UC and CD mucosa. I; inflamed mucosa, N; non-inflamed mucosa

Biopsies from inflamed colonic IBD and UC mucosa expressed significant lower levels of *MAGI3*, *PTEN*, and *TJP1*, compared to non-inflamed colonic mucosa from the same groups of patients (Table 4). There was no overlap between the expression levels of *PTEN* in inflamed colonic mucosa of CD patients compared to non-inflamed CD mucosa (Fig. 3). Furthermore, biopsies from inflamed colonic IBD mucosa expressed a significantly higher level of *PTPN22*, compared to non-inflamed colonic IBD mucosa.

**Table 4:** For genes with a significant genetic association to IBD, CD, or UC, gene expression (ΔCt values) was analyzed in relation to presence of inflammation, using logistic regression. ΔCt values are inversely related to gene expression values. The estimates represent the natural logarithm of the odds ratio with a negative value corresponding to increased odds for inflammation, while a positive value corresponds to decreased odds

Gene expression	Single logistic regression			
	*p* value	Estimate	Estimate (95% CI)	Nagelkerke R^2^
*IBD*; *colonic biopsies* ^*a*^				
*F11R*	0.139	1.04	-0.34–2.43	0.05
*MAGI2*	0.115	0.93	-0.22–2.08	0.06
*MAGI3*	1.53×10^-4^	3.38	1.63–5.13	0.52
*PTEN*	6.72×10^-5^	10.25	5.21–15.29	0.69
*PTPN22*	0.005	-1.76	-2.98– -0.54	0.22
*TJP1*	1.56×10^-4^	4.97	2.39–7.55	0.48
*CD*; *ileal biopsies* ^*b*^				
*F11R*	0.768	0.34	-1.92–2.61	0.01
*MAGI2*	0.065	-2.48	-5.11–0.15	0.35
*MAGI3*	0.107	1.97	-0.43–4.36	0.28
*PTEN*	0.343	3.35	-3.58–10.27	0.08
*PTPN22*	0.388	0.56	-0.71–1.82	0.06
*TJP1*	0.230	2.07	-1.84–5.97	0.09
*CD*; *colonic biopsies* ^*c*^				
*F11R*	0.357	1.30	-1.47–4.06	0.06
*MAGI2*	0.118	1.94	-0.49–4.38	0.21
*MAGI3*	0.047	3.01	0.03–5.98	0.39
*PTEN*	^e^	^e^	^e^	^e^
*PTPN22*	0.029	-2.82	-5.35– -0.29	0.45
*TJP1*	0.028	5.13	0.56-9.69	0.50
*UC*; *colonic biopsies* ^*d*^				
*F11R*	0.408	0.70	-0.96–2.35	0.03
*MAGI2*	0.684	0.30	-1.16–1.77	0.01
*MAGI3*	0.003	3.39	1.19–5.59	0.55
*PTEN*	0.002	7.95	2.82–13.08	0.57
*PTPN22*	0.048	-1.54	-3.06– -0.01	0.18
*TJP1*	0.003	4.83	1.61-8.05	0.47

*IBD* inflammatory bowel disease, *CD* Crohn’s disease, *UC* ulcerative colitis

^a^n_non-inflamed_=29 and n_inflamed=_28. ^b^n_non-inflamed_=11 and n_inflamed_=6. ^c^n_non-inflamed_=12 and n_inflamed_=7. ^d^n_non-inflamed_=16 and n_inflamed_=19. ^e^Complete separation and thereby uncertain data (Mann-Whitney *U*-test showed decreased gene expression level in inflamed mucosa, compared to non-inflamed mucosa; *p*=0.0004)

#### Gene expression versus phenotype

The gene expression levels of analyzed genes were not different between any of the phenotypes (CD, UC, or non-IBD) (Additional file 3: Table S3).

#### Gene expression versus medication

Expression of investigated genes in non-inflamed and inflamed IBD biopsy specimens was not affected by ongoing medical treatment (thiopurines, aminosalicylates, or glucocorticosteroids) (Additional file 4: Table S4). Too few individuals underwent anti-TNF-$\alpha$-antibody treatment for meaningful statistical analysis.

## Discussion

IBD has been associated with an increased paracellular permeability of the intestinal epithelium and a dysfunctional barrier is considered important in the pathogenesis of IBD [6, 7]. The TJ structure is critical for the paracellular barrier properties [9]. Although a few IBD susceptibility markers have been linked to barrier related genes, e.g. *CLDN2* and *MAGI2* [11, 12, 20], the extent to which a dysfunctional barrier is a consequence of genetic factors remains unclear.

The large numbers of susceptibility loci identified in relation to IBD [3–5] only explain 10.9% and 7.7% of the heritability of CD and UC, respectively [3]. Regional heterogeneity of allele frequencies among different sub-populations has been observed [4, 5, 21], and indicates the importance of identifying associations in homogenous populations to increase study power [21] and to explain the genetic variance in IBD. To further clarify the role of the paracellular barrier in the etiology of IBD, a Swedish population of IBD patients and non-IBD controls was genotyped for several TJ related genes and analyzed for expression in the intestinal mucosa.

One strong association was observed between the T-allele of the *MAGI3* SNP marker rs1343126 and IBD, CD, and UC. Further, the C-allele of another *MAGI3* SNP marker (rs6689879) was found to be significantly associated with UC. Presence of this marker also resulted in an increased *MAGI3* expression in ileal non-IBD mucosa, which might contribute to the restriction of inflammation in UC to the colon. To the best of our knowledge we have for the first time demonstrated an association between *MAGI3* and IBD. However, Jostins et al. have previously demonstrated association between this genetic region and CD, but primarily highlighted *PTPN22* [5].

In our study, carriage of at least one IBD associated allele at rs1343126 was nominally associated to a decreased colonic expression of *PTPN22* (located in the same genetic region as *MAGI3*) in the non-IBD subgroup. The corresponding relationship was not identified in inflamed or non-inflamed mucosa of IBD patients. In fact, all relationships between gene expression levels and the susceptibility alleles were observed in mucosal biopsies from non-inflamed, non-IBD controls. Even though the expression of several genes were affected in inflamed mucosa, it is unlikely that genotype – gene expression relationships were concealed in the IBD patients by clinical or subclinical inflammation in the non-inflamed biopsies, since these biopsies were obtained from IBD mucosa that were assessed as histopathologically normal. It is, however, possible that the investigated genes were affected by other factors (e.g. microRNA) that persist in non-inflamed IBD-mucosa [22–24].

Jostins et al. previously identified the C-allele of rs6679677 (*MAGI3*-*PTPN22*) as a susceptibility allele of CD, but a protective allele with respect to UC [5], which was not confirmed by our study. Nevertheless, it is possible that our study missed a possible association due to a comparatively small sample size.

Significant associations were observed between an SNP marker in *MAGI2* (rs6962966) and IBD, CD, as well as UC. Wapenaar et al. [11] previously described an association between this marker and UC. Their study revealed, however, an association to the opposite allele (G) compared to our study (A). Presently we have no explanation for this discrepancy.

In our association study we applied a significance level of *p* < 0.05, and the findings were further followed up by analyzing for a genotype-gene expression correlation in a second independent cohort. However, even if we apply a more stringent *p*-value, by making a Bonferroni adjustment based on the number of analyzed genes (*n* = 8; *p* < 0.006), both *MAGI3* (rs1343126) and *MAGI2* (rs6962966) would still remain significantly associated to IBD.

One limitation of our study was that *MAGI1* and *MAGI3* were only investigated for SNP markers of exons and exon-intron-boundaries, due to the large size of these genes, and that the selection of SNP markers of *MAGI2*, *MAGI3*-*PTPN22*, *PARD3*, and *TJP1* were based on reports in the literature.

*MAGI3* was expressed to a lower level in inflamed, compared to non-inflamed, colonic mucosa from IBD and UC patients. MAGI3 is involved in suppression of the PI3K/Akt pathway due to interaction with PTEN [25], and also in the Wnt/β-catenin pathway [26, 27]. T cells with a high β-catenin level induce T cell activation and intestinal inflammation [28]. Thus, regulation of these pathways might constitute a mechanism by which decreased *MAGI3* expression promotes intestinal inflammation. Furthermore, the *PTPN22* gene expression was shown to be increased in inflamed colonic IBD mucosa, compared to non-inflamed mucosa, which corroborates previous results reported by Chen et al. [29]. The *PTPN22* gene is expressed at a higher level in immune cells compared to non-immune cells [30], and further PTPN22 negatively regulates T cell receptor signaling [31]. In our study gene expression was determined using biopsies, and therefore we are unable to determine the exact cellular origin of the increased expression in inflamed mucosa. It may, however, be due to increased quantities of *PTPN22*-expressing immune cells during inflammation.

Decreased *PTEN* expression was observed in inflamed colonic mucosa of IBD, CD, and UC patients, compared to non-inflamed mucosa. Furthermore, there was no overlap between the *PTEN* expression levels in inflamed colonic mucosa of CD patients, compared to non-inflamed CD mucosa, suggesting *PTEN* as a marker for inflammatory response in colonic CD mucosa. PTEN is described to play a role in the regulation of intestinal permeability [32, 33], and is also defined as a tumor suppressor molecule inhibiting inflammatory response, cell migration, and proliferation via PI3K/Akt pathway [34, 35]. An increased level of microRNA-21 has been described in colonic biopsies from UC patients, compared to normal colonic mucosa, and expression of microRNA-21 in the Caco-2 cell line, a colonic epithelial cell model, resulted in an increased permeability [36]. The mechanism of microRNA-21 has been investigated in Caco-2 cells, and may involve an effect on paracellular permeability via the PTEN level and Akt phosphorylation [33]. Moreover, activation of PI3K/Akt pathway has been demonstrated to inhibit the differentiation of T cells towards regulatory T cells [37] and previously a decreased *PTEN* expression level was observed in intestinal mucosal lymphocytes in CD patients, compared to controls [38].

The decreased *TJP1* expression level observed in inflamed IBD and UC mucosa, compared to non-inflamed mucosa, is consistent with a loss of ZO-1 protein (encoded by *TJP1*) in dextran sulfate sodium induced colitis in mice [39]. Poritz et al. further observed a correlation between loss of ZO-1 and increased paracellular permeability [39].

Gene expression was not affected by ongoing medical treatment (thiopurines, aminosalicylates, or glucocorticosteroids), irrespective of the presence of inflammation. Nevertheless, other studies have shown that infliximab, an anti-TNF-antibody, can impact the expression of genes associated with intestinal permeability [40] and mucosal cytokine expression [41]. Among our patients, too few were treated with infliximab to allow meaningful analysis with this drug.

## Conclusions

In conclusion, our findings support a genetic basis for modulation of the intestinal epithelial barrier in IBD, and point to a complex regulation of TJ gene expression through a secondary role of inflammation. By focusing on a Swedish population and a network of interacting TJ components we have identified *MAGI3* as a new candidate gene for IBD.

## Additional files

Additional file 1: Table S1. Single nucleotide polymorphism markers included in the study. (XLSX 16 kb)

Additional file 2: Table S2. Inflammatory bowel disease-phenotypes were investigated by performing case–control approach in genetic association studies. (XLSX 24 kb)

Additional file 3: Table S3. For genes with a significant genetic association to IBD, CD, or UC, gene expression was analyzed in relation to phenotype, using logistic regression. (DOCX 16 kb)

Additional file 4: Table S4. For genes with a significant genetic association to IBD, CD, or UC, gene expression was analyzed in relation to medical treatment, using logistic regression. (DOCX 17 kb)

## Abbreviations

CD: Crohn’s disease
C_T_: Threshold cycle
Fc: Fold change
IBD: Inflammatory bowel disease
OR: Odds ratio
RQ: Relative quantification
SNP: Single nucleotide polymorphism
TJ: Tight junctions
UC: Ulcerative colitis
