# Supplementary material for: Genetic variation and expression levels of tight junction genes identifies association between MAGI3 and inflammatory bowel disease
Source: BMC Gastroenterol. 2017 May 25;17:68. doi: 10.1186/s12876-017-0620-y (PMC5445404; doi:10.1186/s12876-017-0620-y)
Supplement: Supplementary file 3 — For genes with a significant genetic association to IBD, CD, or UC, gene expression was analyzed in relation to phenotype, using logistic regression. (DOCX 16 kb) [file 12876_2017_620_MOESM3_ESM.docx]

**Additional file 3: Table S3.** For genes with a significant genetic association to IBD, CD, or UC, gene expression (ΔCt values) was analyzed in relation to phenotype, using logistic regression. ΔCt values are inversely related to gene expression values. The estimates represent the natural logarithm of the odds ratio with a negative value corresponding to increased odds, while a positive value corresponds to decreased odds.

| Gene expression | Single logistic regression | | |  | Single logistic regression | | |  |
| --- | --- | --- | --- | --- | --- | --- | --- | --- |
|  | *p* value | Estimate | Estimate (95% CI) | Nagelkerke R^2^ | *p* value | Estimates | Estimate (95% CI) | Nagelkerke R^2^ |
|  | |  |  |  |  |  |  |  |
| **Non-inflamed ileal mucosa** | |  |  |  | **Non-inflamed colonic mucosa** | | |  |
| ***CD vs. non-IBD^a^*** | |  |  |  | ***CD vs. non-IBD^a^*** | |  |  |
| *F11R* | 0.141 | -1.22 | -2.85-0.41 | 0.12 | 0.086 | -2.06 | -4.42-0.29 | 0.14 |
| *MAGI2* | 0.141 | 1.29 | -0.43-3.00 | 0.11 | 0.732 | -0.38 | -2.53-1.77 | <0.01 |
| *MAGI3* | 0.119 | -0.92 | -2.08-0.24 | 0.13 | 0.776 | -0.17 | -1.36-1.02 | <0.01 |
| *PTEN* | 0.416 | -1.64 | -5.58-2.31 | 0.03 | 0.770 | -0.58 | -4.45-3.29 | <0.01 |
| *PTPN22* | 0.094 | 1.20 | -1.21-2.61 | 0.14 | 0.212 | 1.04 | -0.60-2.68 | 0.07 |
| *TJP1* | 0.334 | -0.86 | -2.61-0.88 | 0.05 | 0.501 | -0.94 | -3.67-1.79 | 0.02 |
|  |  |  |  |  |  |  |  |  |
| ***UC vs. non-IBD^b^*** | |  |  |  | ***UC vs. non-IBD^b^*** | |  |  |
| *F11R* | 0.057 | -1.85 | -3.76-0.06 | 0.22 | 0.120 | -1.49 | -3.37-0.39 | 0.10 |
| *MAGI2* | 0.307 | 0.91 | -0.84-2.66 | 0.05 | 0.033 | 2.55 | 0.20-4.90 | 0.21 |
| *MAGI3* | 0.053 | -1.27 | -2.55-0.01 | 0.20 | 0.248 | -0.75 | -2.03-0.52 | 0.06 |
| *PTEN* | 0.016 | -4.62 | -8.38- -0.85 | 0.33 | 0.678 | -0.64 | -3.68-2.39 | <0.01 |
| *PTPN22* | 0.389 | 0.71 | -0.90-2.31 | 0.03 | 0.149 | 1.04 | -0.37-2.45 | 0.09 |
| *TJP1* | 0.129 | -1.24 | -2.85-0.36 | 0.12 | 0.181 | -1.63 | -4.03-0.76 | 0.07 |
|  |  |  |  |  |  |  |  |  |
| ***UC vs. CD^c^*** | |  |  |  | ***UC vs. CD^c^*** |  |  |  |
| *F11R* | 0.505 | -0.66 | -2.62-1.29 | 0.03 | 0.844 | 0.19 | -1.73-2.11 | <0.01 |
| *MAGI2* | 0.654 | -0.37 | -1.99-1.25 | 0.01 | 0.025 | 3.46 | 0.44-6.49 | 0.30 |
| *MAGI3* | 0.677 | -0.31 | -1.75-1.14 | 0.01 | 0.378 | -0.69 | -2.21-0.84 | 0.04 |
| *PTEN* | 0.042 | -5.17 | -10.15- -0.19 | 0.29 | 0.886 | -0.27 | -4.01-3.47 | <0.01 |
| *PTPN22* | 0.355 | -0.62 | -1.93-0.69 | 0.05 | 0.827 | 0.16 | -1.23-1.54 | <0.01 |
| *TJP1* | 0.416 | -0.93 | -3.16-1.31 | 0.04 | 0.526 | -0.82 | -3.34-1.70 | 0.02 |
|  |  |  |  |  |  |  |  |  |
| **Inflamed ileal mucosa** | |  |  |  | **Inflamed colonic mucosa** | | |  |
| ***UC vs. CD^d^*** |  |  |  |  | ***UC vs. CD^d^*** |  |  |  |
| *F11R* |  |  |  |  | 0.965 | -0.05 | -2.35-2.24 | <0.01 |
| *MAGI2* |  |  |  |  | 0.963 | 0.04 | -1.54-1.61 | <0.01 |
| *MAGI3* |  |  |  |  | 0.618 | 0.44 | -1.30-1.39 | 0.12 |
| *PTEN* |  |  |  |  | 0.153 | -3.75 | -8.89-1.39 | 0.12 |
| *PTPN22* |  |  |  |  | 0.122 | 1.50 | -0.40-3.40 | 0.14 |
| *TJP1* |  |  |  |  | 0.560 | -0.86 | -3.74-2.03 | 0.02 |

^a^Ileal mucosa: n_CD_=11, n_non-IBD_=24. Colonic mucosa: n_CD_=12, n_non-IBD_=33. ^b^Ileal mucosa: n_UC_=12, n_non-IBD_=24. Colonic mucosa: n_UC_=16, n_non-IBD_=33. ^c^Non-inflamed ileal mucosa; n_UC_=12, n_CD_=11. Non inflamed colonic mucosa; n_UC_=16, n_CD_=12. ^d^Inflamed colonic mucosa; n_UC_=19, n_CD_=7.

IBD: inflammatory bowel disease, CD: Crohn’s disease, UC: ulcerative colitis, non-IBD: non-inflamed non-IBD controls.
