# Supplementary material for: Genetic variation and expression levels of tight junction genes identifies association between MAGI3 and inflammatory bowel disease
Source: BMC Gastroenterol. 2017 May 25;17:68. doi: 10.1186/s12876-017-0620-y (PMC5445404; doi:10.1186/s12876-017-0620-y)
Supplement: Supplementary file 4 — For genes with a significant genetic association to IBD, CD, or UC, gene expression was analyzed in relation to medical treatment, using logistic regression. (DOCX 17 kb) [file 12876_2017_620_MOESM4_ESM.docx]

**Additional file 4: Table S4.** For genes with a significant genetic association to IBD, CD, or UC, gene expression (ΔCt values) was analyzed in relation to medical treatment, using logistic regression. ΔCt values are inversely related to gene expression values. The estimates represent the natural logarithm of the odds ratio with a negative value corresponding to increased odds, while a positive value corresponds to decreased odds.

| Gene expression | Single logistic regression | | |  | Single logistic regression | | |  |
| --- | --- | --- | --- | --- | --- | --- | --- | --- |
|  | *p* value | Estimate | Estimate (95% CI) | Nagelkerke R^2^ | *p* value | Estimates | Estimate (95% CI) | Nagelkerke R^2^ |
| **Non-inflamed IBD ileum** | |  |  |  | **Non-inflamed IBD colon** | | |  |
| ***Thiopurine (n=7) vs. no thiopurines (n=17)*** | |  |  |  | ***Thiopurine (n=7) vs. no thiopurines (n=22)*** | | |  |
| *F11R* | 0.358 | 0.98 | -1.11-3.07 | 0.05 | 0.214 | -1.64 | -4.24-0.95 | 0.09 |
| *MAGI2* | 0.931 | 0.08 | -1.66-1.81 | <0.01 | 0.580 | 0.66 | -1.68-3.00 | 0.02 |
| *MAGI3* | 0.752 | -0.23 | -4.17-3.41 | <0.01 | 0.139 | -1.46 | -3.39-0.47 | 0.13 |
| *PTEN* | 0.844 | -0.38 | -4.17-3.41 | <0.01 | 0.553 | 1.27 | -2.93-5.48 | 0.02 |
| *PTPN22* | 0.703 | 0.266 | -1.10-1.63 | <0.01 | 0.819 | -0.19 | -1.78-1.41 | <0.01 |
| *TJP1* | 0.160 | 1.74 | -0.69-4.18 | 0.13 | 0.412 | 1.16 | -1.61-3.93 | 0.03 |
|  |  |  |  |  |  |  |  |  |
| ***Aminosalicylate (n=13) vs. no aminosalicylate (n=11)*** | |  |  |  | ***Aminosalicylate (n=14) vs. no aminosalicylate (n=15)*** | | |  |
| *F11R* | 0.764 | 0.27 | -1.51-2.06 | <0.01 | 0.784 | 0.26 | -1.62-2.15 | <0.01 |
| *MAGI2* | 0.333 | 0.88 | -0.90-2.65 | 0.06 | 0.333 | 1.05 | -1.08-3.19 | 0.04 |
| *MAGI3* | 0.840 | -0.14 | -1.46-1.19 | <0.01 | 0.985 | 0.01 | -1.39-1.42 | <0.01 |
| *PTEN* | 0.372 | -1.63 | -5.22-1.96 | 0.05 | 0.257 | 2.28 | -1.66-6.21 | 0.06 |
| *PTPN22* | 0.181 | 0.93 | -0.43-2.30 | 0.10 | 0.655 | -0.32 | -1.70-1.07 | <0.01 |
| *TJP1* | 0.600 | -0.53 | -2.51-1.45 | 0.02 | 0.477 | 0.91 | -1.59-3.40 | 0.02 |
|  |  |  |  |  |  |  |  |  |
| ***Corticosteroid (n=7) vs. no corticosteroid (n=17)*** | |  |  |  | ***Corticosteroid (n=8) vs. no corticosteroid (n=21)*** | | |  |
| *F11R* | 0.375 | -0.91 | -2.91-1.10 | 0.05 | 0.819 | 0.25 | -1.85-2.34 | <0.01 |
| *MAGI2* | 0.143 | 1.41 | -0.48-3.29 | 0.14 | 0.961 | 0.06 | -2.20-2.31 | <0.01 |
| *MAGI3* | 0.861 | -0.13 | -1.57-1.32 | <0.01 | 0.976 | -0.02 | -1.60-1.55 | <0.01 |
| *PTEN* | 0.742 | -0.64 | -4.45-3.17 | <0.01 | 0.815 | -0.50 | -4.70-3.70 | <0.01 |
| *PTPN22* | 0.110 | 1.27 | -0.29-2.83 | 0.16 | 0.535 | 0.50 | -1.07-2.06 | 0.02 |
| *TJP1* | 0.930 | 0.10 | -2.04-2.24 | <0.01 | 0.972 | -0.05 | -2.78-2.65 | <0.01 |
|  |  |  |  |  |  |  |  |  |
| **Inflamed IBD ileum^a^** | |  |  |  | **Inflamed colonic mucosa** | | |  |
| ***Thiopurine (n=1) vs. no thiopurines (n=5)*** | |  |  |  | ***Thiopurine (n=9) vs. no thiopurines (n=19)*** | | |  |
| *F11R* |  |  |  |  | 0.709 | -0.40 | -2.49-1.69 | <0.01 |
| *MAGI2* |  |  |  |  | 0.273 | 0.86 | -0.67-2.39 | 0.06 |
| *MAGI3* |  |  |  |  | 0.242 | -1.04 | -2.79-0.70 | 0.08 |
| *PTEN* |  |  |  |  | 0.197 | 2.78 | -1.44-7.00 | 0.09 |
| *PTPN22* |  |  |  |  | 0.375 | 0.77 | -0.93-2.48 | 0.04 |
| *TJP1* |  |  |  |  | 0.250 | 1.65 | -1.16-4.46 | 0.07 |
|  |  |  |  |  |  |  |  |  |
| ***Aminosalicylate (n=1) vs. no aminosalicylate (n=5)*** | |  |  |  | ***Aminosalicylate (n=15) vs. no aminosalicylate (n=13)*** | | |  |
| *F11R* |  |  |  |  | 0.287 | -1.11 | -3.15-0.93 | 0.06 |
| *MAGI2* |  |  |  |  | 0.596 | 0.36 | -0.98-1.71 | 0.01 |
| *MAGI3* |  |  |  |  | 0.144 | -1.28 | -3.00-0.44 | 0.12 |
| *PTEN* |  |  |  |  | 0.484 | -1.37 | -5.20-2.46 | 0.02 |
| *PTPN22* |  |  |  |  | 0.866 | 0.13 | -1.38-1.64 | <0.01 |
| *TJP1* |  |  |  |  | 0.682 | -0.53 | -3.08-2.02 | <0.01 |
|  |  |  |  |  |  |  |  |  |
| ***Corticosteroid (n=1) vs. no corticosteroid (n=5)*** | |  |  |  | ***Corticosteroid (n=12) vs. no corticosteroid (n=16)*** | | |  |
| *F11R* |  |  |  |  | 0.880 | -0.15 | -2.11-1.81 | <0.01 |
| *MAGI2* |  |  |  |  | 0.929 | 0.06 | -1.28-1.41 | <0.01 |
| *MAGI3* |  |  |  |  | 0.256 | -0.93 | -2.54-0.68 | 0.07 |
| *PTEN* |  |  |  |  | 0.822 | -0.44 | -4.23-3.36 | <0.01 |
| *PTPN22* |  |  |  |  | 0.859 | -0.14 | -1.65-1.38 | <0.01 |
| *TJP1* |  |  |  |  | 0.290 | -1.48 | -4.23-1.26 | 0.06 |

^a^There were too few inflamed ileal biopsies for meaningful statistical analysis.

IBD: inflammatory bowel disease, CD: Crohn’s disease, UC: ulcerative colitis.
